# Systematic comparison of two whole-genome amplification methods for targeted next-generation sequencing using frozen and FFPE normal and cancer tissues

**DOI:** 10.1038/s41598-017-04419-9

**Published:** 2017-06-22

**Authors:** Pedro Mendez, Li Tai Fang, David M. Jablons, Il-Jin Kim

**Affiliations:** 1 Thoracic Oncology Laboratory, Department of Surgery, University of California San Francisco, San Francisco, CA USA; 2 Comprehensive Cancer Center, University of California San Francisco, San Francisco, CA USA

## Abstract

Sequencing key cancer-driver genes using formalin-fixed, paraffin-embedded (FFPE) cancer tissues is becoming the standard for identifying the best treatment regimen. However, about 25% of all samples are rejected for genetic analyses for reasons that include too little tissue to extract enough high quality DNA. One way to overcome this is to do whole-genome amplification (WGA) in clinical samples, but only limited studies have tested different WGA methods in FFPE cancer specimens using targeted next-generation sequencing (NGS). We therefore tested the two most commonly used WGA methods, multiple displacement amplification (MDA-Qiagen REPLI-g kit) and the hybrid or modified PCR-based method (Sigma/Rubicon Genomics Inc. GenomePlex kit) in FFPE normal and tumor tissue specimens. For the normalized copy number analysis, the FFPE process caused none or very minimal bias. Variations in copy number were minimal in samples amplified using the GenomePlex kit, but they were statistically significantly higher in samples amplified using the REPLI-g kit. The pattern was similar for variant allele frequencies across the samples, which was minimal for the GenomePlex kit but highly variable for the REPLI-g kit. These findings suggest that each WGA method should be tested thoroughly before using it for clinical cancer samples.

## Introduction

The advent of the polymerase chain reaction (PCR) for genetic analyses^1–3^ has led to significant progress in several fields, including forensic science, drug selection, and molecular diagnoses of diseases such as cancer^4–8^. However, most of these PCR-based applications require at least nanogram level of DNA to robustly amplify and analyze original DNA^9–11^, which is not available from every biological or clinical sample. A new technology, whole genome amplification (WGA)^12, 13^, was designed to address this problem. WGA methods can be classified into three major categories. One of the earliest method is the degenerate oligonucleotide primed (DOP)-PCR, which uses random priming with thermostable polymerases, resulting in uniform amplification. Although (DOP)-PCR is relatively easy to use, there are also potential drawbacks, such as increase of amplification error (false positive rate) and reduced genome coverage^10, 12, 13^. The second category is multiple displacement amplification (MDA). This method does random priming using phi29 DNA polymerase in an isothermal amplification of the template DNA^10, 14–16^. Phi29 DNA polymerase has high fidelity, resulting in a low error rate and strand displacement activity^10^. This isothermal exponential amplification is known to have relatively low false-positive and false-negative rates and higher genome coverage, but has a skewed amplification pattern and overrepresentation of the amplified region^10^. The most commonly used MDA-based kit is REPLI-g (Qiagen). The last category is a hybrid amplification method using both isothermal and then PCR-based amplification^10^. The best examples are Multiple Annealing and Looping Based Amplification Cycles (MALBAC) and Rubicon Genomics Inc.’s technologies, such as PicoPLEX and GenomePlex (Sigma-Aldrich)^9, 10, 16^. These hybrid methods increase the amplification uniformity while keeping reasonably low false positive and negative rates^10^.

These different WGA methods have advantages and disadvantages in different applications^9–11, 16^. Most WGA evaluations have been done in single cells^10, 11, 17, 18^ and for copy number analyses^9, 17–19^. Some studies also tested other biological specimens such as cancer formalin-fixed, paraffin-embedded (FFPE) DNA mainly for copy number^19, 20^. Though some^21^ assessed variation or mutation detection using cancer FFPE samples. To select the best treatment regimens for patients with cancer, it is becoming more important to know the mutation status of key genes (i.e. *EGFR* and *EML4-ALK* for lung adenocarcinoma patients)^22–24^. Most mutation and genetic analyses of clinical samples are done in a Clinical Laboratory Improvement Amendments (CLIA) laboratory using cancer FFPE biopsy specimens^25^. Around 25% of samples sent to CLIA laboratories are rejected for molecular analyses because the number of tumor cells or amount of tissue in the specimens is inadequate for DNA extraction^26^. Many next-generation sequencing (NGS) CLIA laboratories use targeted NGS panels with selected genes rather than whole exome or genome sequencing panels^25^. Most published evaluations of WGA on cancer specimens are focused on large NGS panels like exome or whole genome^10^. Although evaluating the exome or whole genome would provide a comprehensive overview for the functional assessment of WGA, more and more targeted sequencing approaches using a small panel with around 10–500 genes are becoming popular for patient screening in CLIA labs^25, 27–29^. Therefore, it is important that different WGA methods using the targeted NGS panels should be evaluated in clinically challenging samples like FFPE DNA.

In this study, we evaluated the two most commonly used WGA methods, MDA and hybrid amplification^10^, on challenging clinical specimens using a targeted NGS cancer panel with selected genes. The two WGA methods were thoroughly evaluated for DNA yield and quality, library prep result, and the final sequencing status using targeted NGS panels.

## Results

### WGA kits and NGS Quality metrics

The summary of the NGS quality metrics can be found in the Table 1. The number of mapped, on target reads per sample are ranged from 109,547 to 296,996× for the AmpliSeq customized panel (Thermo Fisher Scientific), and from 289,631 to 495,713× for the NextDay Seq panel (CureSeq Inc). The mean sequencing depth was >1,000× in all the samples analyzed. We noticed that the percentage of reads on target for AmpliSeq custom panel was much lower (68.25%) than the NextDay Seq panel (98.54%). The coverage uniformity was >84% in average. When we broke down the analysis by tissue type and WGA kit, we observed that NGS libraries prepared with either REPLI-g or GenomePlex had significantly lower coverage uniformity than non-WGA samples regardless of the tissue type (Figure S1a). In the case of REPLI-g, the coverage uniformity was also significantly lower than GenomePlex (Figure S1a). This phenomenon was independent of the sequencing depth (Figure S1b).

**Table 1:** Sequencing quality metrics.

		Mean of Mapped Reads per Sample	% of Reads On Target	Average of Mapped Reads, On Target per Sample	Mean Depth (On Target)	Coverage Uniformity
**Sample LC#1**						
LC #1 ACP	Replicate #1	190,135	64.45%	122,539	1,152	90.59%
	Replicate #2	160,040	68.63%	109,838	1,044	92.64%
	Replicate #3	191,567	68.23%	130,703	1,232	91.92%
**Sample LC#1**						
LC #2 ACP	Replicate #1	257,591	70.71%	182,132	1,027	84.01%
**Sample EC#1**						
EC #1 NDS	Replicate #1	503,058	98.54%	495,705	5,131	93.90%
EC #1 ACP	Replicate #1	429,481	69.23%	297,322	2,795	89.44%

LC: Lung cancer; ACP: Ampliseq custom panel; NDS: NextDay Seq PAN Cancer panel.

### Variant Allele Frequencies (VAF) Variability Threshold in NGS

One lung cancer FFPE sample was sequenced in triplicates to calculate variations caused by different WGA methods. The three independent sets of libraries were prepared in three different days. Among the 14 germline and 11 somatic variants consistently found in all three independent replicates, we found a standard deviation of the variant allele frequency (VAF) of 0.0316 and 0.0219 in non-WGA FFPE normal and tumor samples, respectively (data not shown). Thus, we concluded that using a VAF threshold of 0.05 to measure the differences in VAF between experimental conditions would be safe and accurate.

### NGS Data Bias Introduced by FFPE procedure

Next, we tested the degree of bias introduced in NGS data, strictly generated by the formalin fixation and paraffin embedding process. We analyzed the gene copy number (GCN) of the non-WGA FFPE DNA samples, using as reference sample of its frozen DNA counterparts in two lung cancer samples sets (LC#1 and LC#2), and AmpliSeq customized DNA panel. The median GCN values in non-WGA FFPE normal and tumor tissues were not statically different in none of both lung cancer samples analyzed (Non-WGA FFPE N vs. frozen N, p = 0.65 for LC#1 and p = 0.32 for LC#2; Non-WGA FFPE T vs. frozen T, p = 0.54 for LC#1 and p = 0.44 for LC#2). No amplicons showed bias increase or decrease in GCN value for the Frozen normal nor tumor sample (data not shown). The correlation of VAF between frozen tumor and frozen normal DNA (slope = 0.78, R^2^ = 0.84) was very similar to the correlation found between VAFs from non-WGA FFPE-T vs. non-WGA FFPE-N tissues (slope = 0.75, R^2^ = 0.73**)**.

### WGA Variability in Frozen Tissue

To grasp the variability of WGA kits in DNA extracted from frozen tissues, we amplify DNA extracted from a paired frozen-FFPE normal and tumor tissues with both WGA kits. Next, NGS libraries were prepared using the AmpliSeq customized panel. When quantifying the NGS libraries, we observed that the yield of the NGS libraries prepared from WGA products derived from frozen tissues were much lower than the yield of the NGS libraries prepared from the same samples but from FFPE origin. In the case of the REPLI-g kit, the yield was insufficient to meet the minimum requirements of the sequencing reaction (Figure S2a), while GenomePlex kit provided enough DNA for the targeted NGS analysis. To rule out any potential issue caused by AmpliSeq library preparation kit, not by WGA method, the NextDay Seq panel was tested too. The results confirmed what we observed with the AmpliSeq panel (Figure S2b). Thus, we could not evaluate the variability of WGA in frozen samples, due to not enough WGA products from REPLI-g FFPE kit.

### NGS Bias caused by GenomePlex and REPLI-g WGA methods

**Figure 1:**
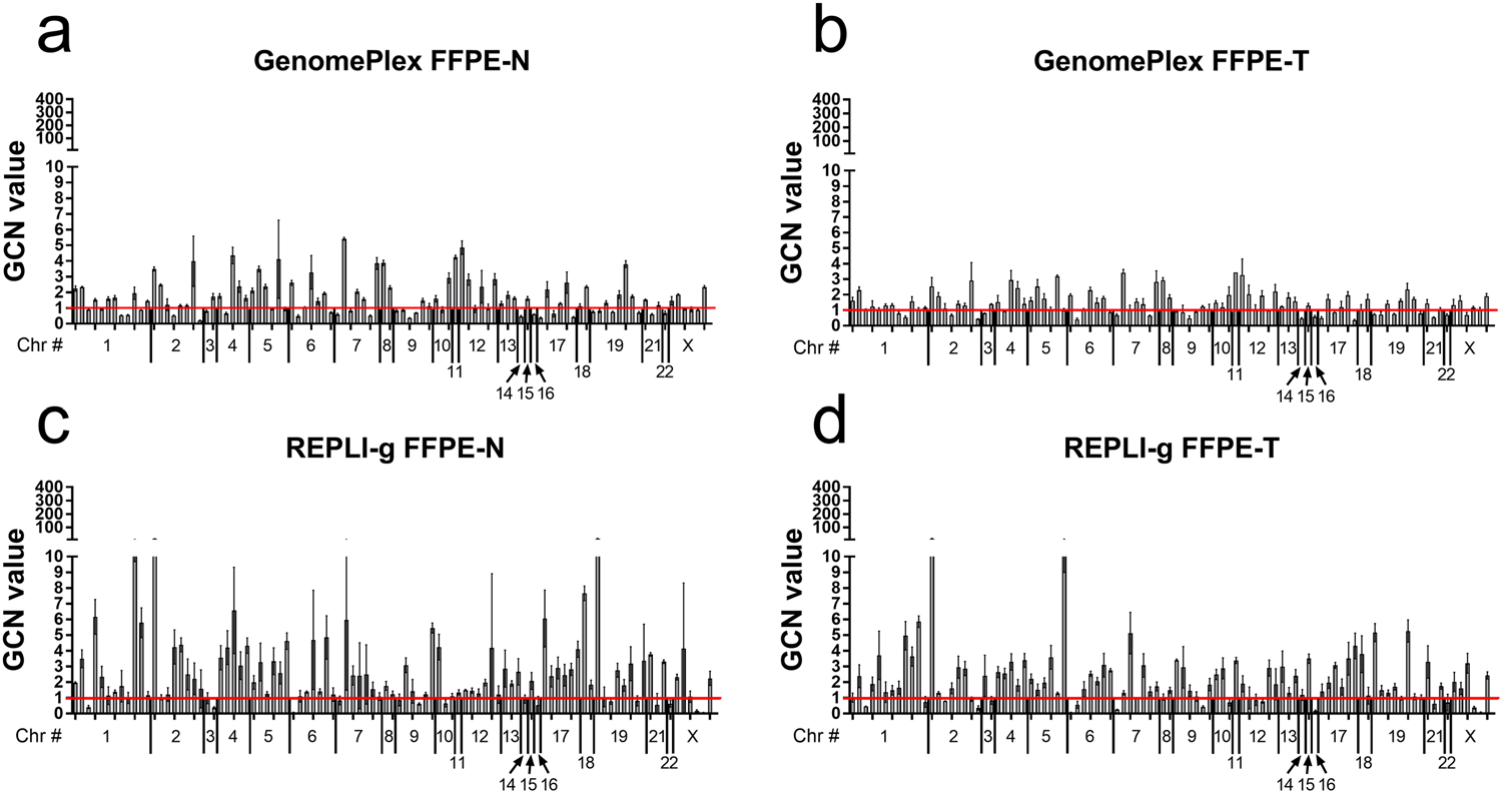
Gene Copy number (GCN) data from GenomePlex and REPLI-g amplified FFPE DNAs. (**a**) Histogram of GCN values for all 97 amplicons of the AmpliSeq customized library preparation panel in the GenomePlex FFPE-N, (**b**) GenomePlex FFPE-T, (**c**) REPLI-g FFPE-N, (**d**) REPLI-g FFPE-T samples. Each bar represents the average of GCN value ± SD of three independent replicates of the library preparation kit experiments. The range of normal gene dosage is 1 ± 0.25. The normalization of GCN data is described in the Methods section. GCN: normalized gene copy number; FFPE: formalin-fixed and paraffin embedded tissue.

**Figure 2:**
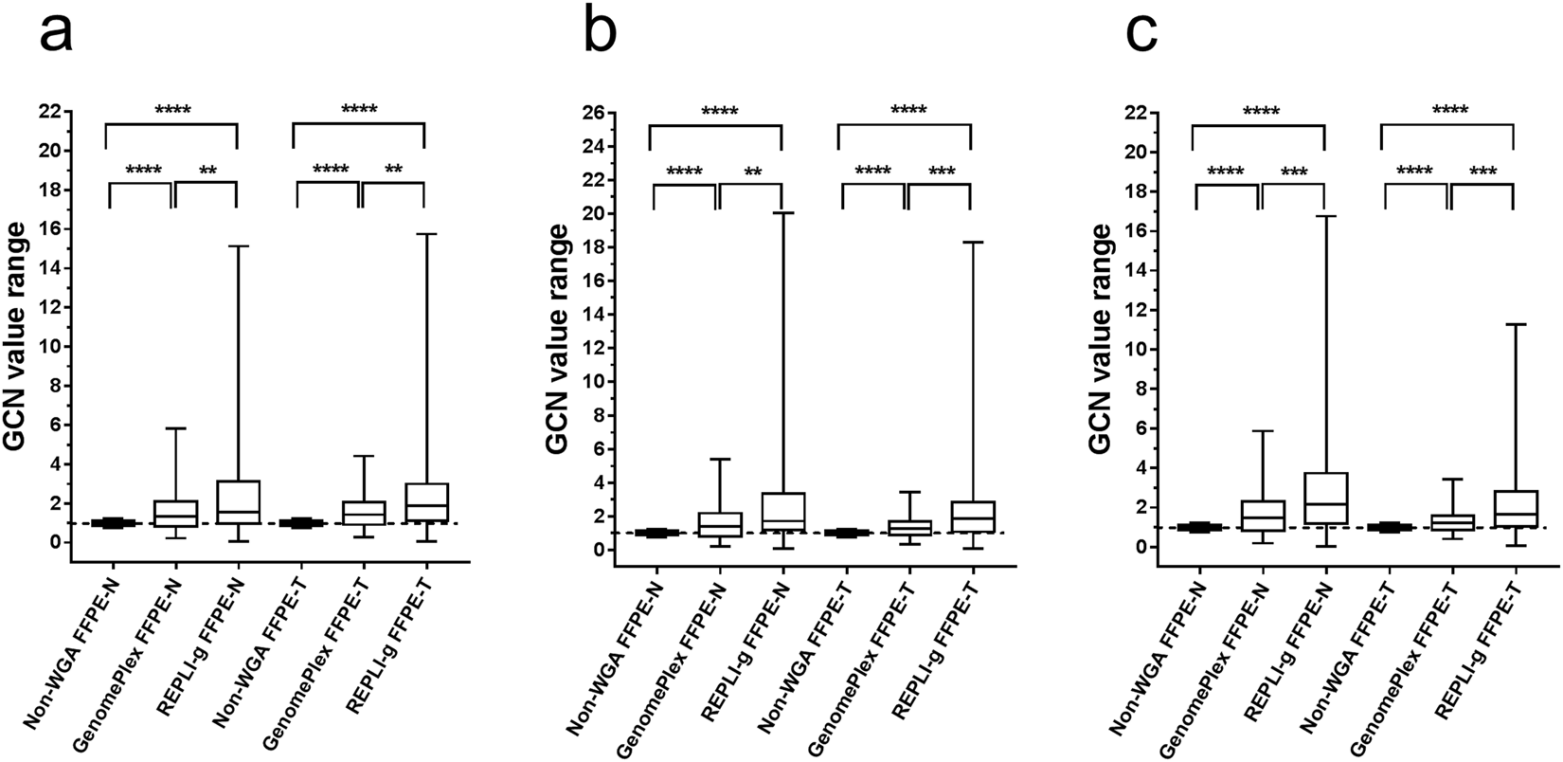
Range of GCN values across all the experimental conditions and technical replicates. Boxplot (min. to max. and median values) of the range of GCN values for all 97 amplicons included in the library reparation of the AmpliSeq customized panel for all the experimental conditions. (**a**) Represents the data from library preparation replicate #1, (**b**) or #2, (**c**) or #3. A two-tailed, Wilcoxon matched-pairs signed rank test was used to assess the statistical significance of the difference between different experimental conditions. The range of normal gene dosage is 1 ± 0.25. *Indicates p < 0.05, **p < 0.01, ***p < 0.001 and ****p < 0.0001. FFPE: formalin-fixed and paraffin embedded tissue, WGA: Whole genome amplification, GCN: Gene copy number.

At the GCN level, the results have consistently shown that both WGA kits introduced significant bias, compared to non-WGA FFPE DNAs. This was confirmed in the triplicate experiments of LC#1 (Fig. 1), in the LC#2 (Figure S3), and in the EC#1 (Figure S4), when the NGS libraries were prepared using the AmpliSeq panel. The GCN values range for REPLI-g FFPE DNAs, across all 97 amplicons, was significantly higher than GenomePlex and also higher than non-WGA FFPE DNAs, regardless of the tissue type analyzed in each triplicate of LC#1 (Fig. 2a–c), as well in the sample LC#2 (Figure S5a), or in EC#1 (Figure S5b). Interestingly, when the GCN data from sample EC#1 was analyzed in NGS libraries prepared with the NextDay Seq panel, REPLI-g only produced a significantly higher bias than GenomPlex in the normal, but not in the tumor tissues (Figures S5c and S6).

Around 60% of the variants found in all three replicates of the non-WGA normal DNA from LC#1 were homozygous or heterozygous. Nonetheless, there were few variants with low VAF, including a haplotype in chromosome 6 with VAF ~0.25, and three more SNVs with VAF < 0.15 (data not shown). This data suggests that although we selected phenotypically normal tissue (adjacent to tumor tissue), it may contain some somatic mutations as previously reported^30–32^.

**Figure 3:**
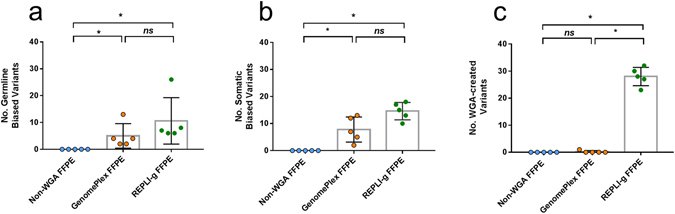
Number of biased variants caused by WGA kits in all samples. Histograms of mean ± SD of the number of biased (**a**) germline, (**b**) somatic and (**c**) WGA-created variants. The data includes the three replicates of lung cancer #1, as well as the lung cancer #2 and esophageal cancer #1 sets. A two-tailed Mann-Whitney test (comparing ranks) was used for analysis. *Indicates p < 0.05 and **p < 0.01. FFPE: formalin-fixed and paraffin embedded tissue, WGA: Whole genome amplification.

As for the bias introduced by WGA methods at VAF level, first we analyzed how many germline and/or somatic variants shifted their VAF ( ± 0.05), compared to their non-WGA FFPE tissue pairs. Second, we studied how many false positive variants were created during the WGA process (non-detected in non-WGA FFPE samples). Among all the sample sets analyzed with the AmpliSeq customized panel, REPLI-g had significantly higher number of altered germline (Fig. 3a), somatic (Fig. 3b), and WGA-created variants (Fig. 3c) than non-WGA FFPE samples. In average, REPLI-g double to GenomePlex result in the number of altered germline (10.6 vs 5; Fig. 3a) and somatic variants (14.6 vs. 7.8; Fig. 3b) per sample or replicate. However, the biggest difference was observed in the number of false positive variants that REPLI-g created during the WGA procedure. GenomePlex created an average of 0.2 variants per sample/replicate, while REPLI-g generated 28 variants (Fig. 3c; p = 0.0079). In the sub-analysis of the three replicates of the LC#1, REPLI-g created a significantly higher number of variants per replicate during WGA (26 vs. 0; p < 0.001; data not shown). Yet, the number of biased germline (6.3 vs. 3.3; p = 0.1) or somatic variants (14.0 vs. 10.7; p = 0.29), although higher in REPLI-g, did not reach the statistical significance (data not shown).

**Figure 4:**
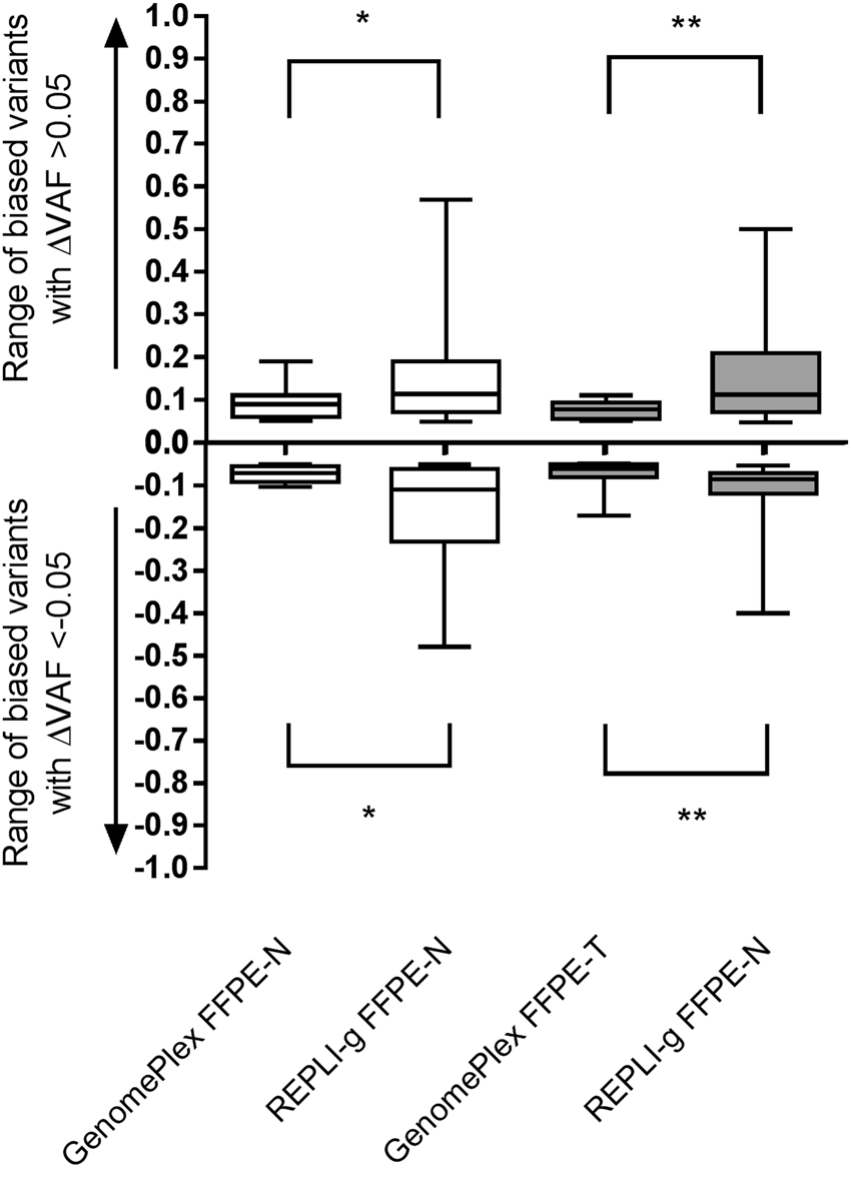
Range of biased VAF values caused by WGA kits. Boxplot (min. to max. and median values) of the biased VAF values. One variant was considered biased, when its VAF was ± 0.05 of the VAF value of the same variant in its reference samples (non-WGA FFPE DNAs). The data includes the values from the independent triplicates of library preparations from lung cancer #1, the lung cancer #2, and esophageal cancer #1. A two-tailed Mann-Whitney test (comparing ranks) test was used to assess the statistical significance of the difference between different experimental conditions. *Indicates p < 0.05 and **p < 0.01. FFPE: formalin-fixed and paraffin embedded tissue, WGA: Whole genome amplification.

In addition to the difference in number of biased variants, we also wanted to understand if the mean of VAF values were significantly different between both WGA kits. We defined ΔVAF as the difference between the VAF value of a given variant in any WGA kit and the VAF of the same variant in the non-WGA FFPE-paired sample. REPLI-g always had a significant large ΔVAF compare to GenomePlex, regardless of the tissue type (normal or tumor) or whether the bias value was positive (>0.05) or negative (<−0.05; Fig. 4).

### Mechanism of VAF Bias by WGA kits

**Figure 5:**
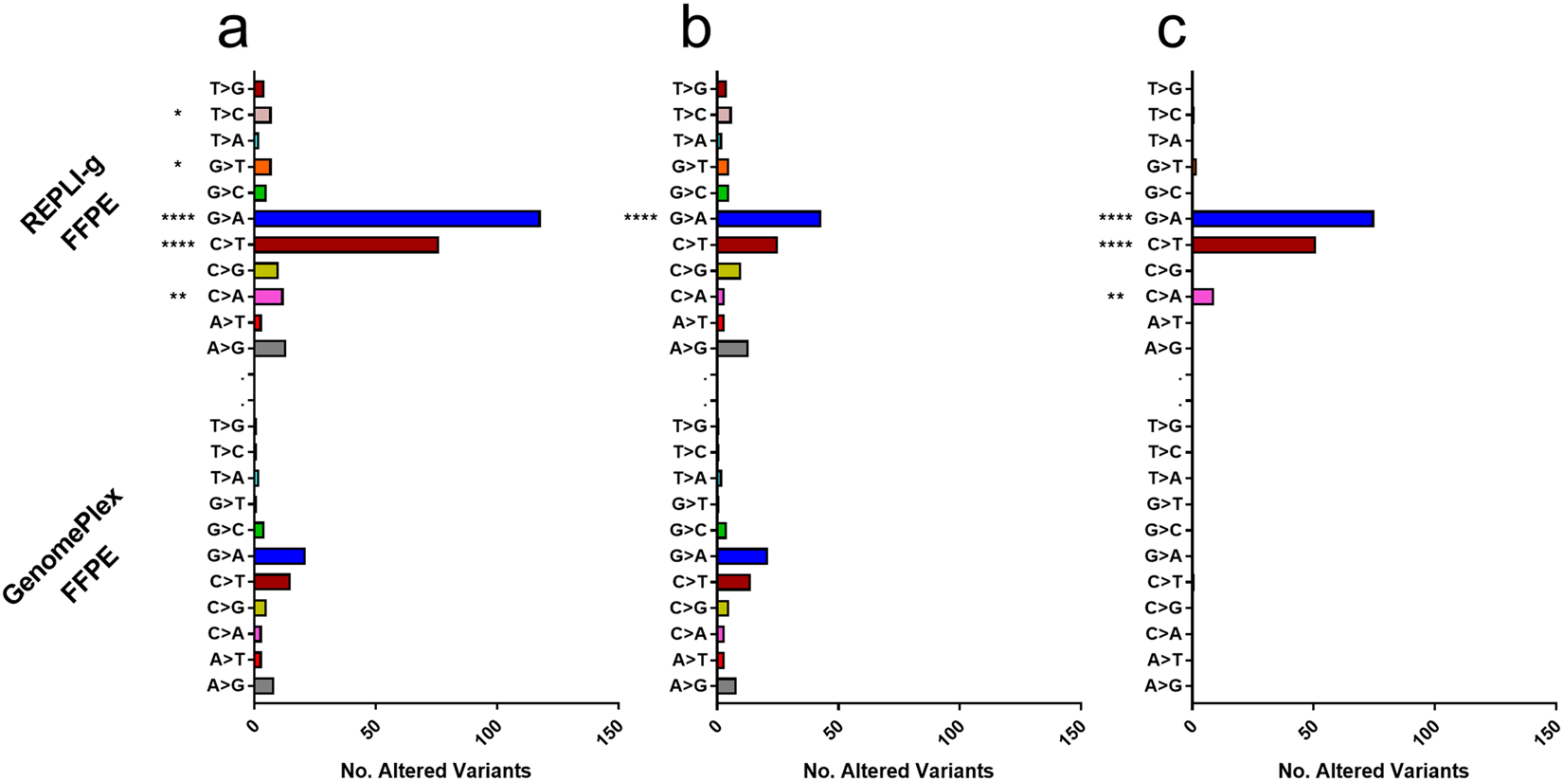
Biased variants analyzed by nucleotide base substitution, variant type, and the used WGA kit. Histograms of number of variants biased in (**a**) all the altered variants, (**b**) germline plus somatic variants or (**c**) on false-positive variants created during WGA. The data includes the three replicates of lung cancer #1, as well as the lung cancer #2 and esophageal cancer #1 sets. The statistical significance was calculated as described in methods. *Indicates p < 0.05, **p < 0.01, ***p < 0.001 and ****p < 0.0001. FFPE: formalin-fixed and paraffin embedded tissue, WGA: Whole genome amplification.

Taking advantage of the technical triplicates of sample LC#1, we studied if there were specific genomic positions that were more prone to cause biases by WGA kits. On the analysis of biased variants, by nucleotide, we observed that cytosine had significantly higher number of variants than adenine (56 vs. 16; p < 0.0001) and thymine (56 vs. 12; p < 0.0001). Guanine also had higher number of variants than adenine (77 vs. 16; p < 0.0001) and thymine (77 vs. 12; p < 0.0001). Next, we broke down the analysis by nucleotide substitution type to see if there was any particular substitution associated with higher prevalence or WGA kit. Among all the variants analyzed, five substitutions had significantly higher number of variations in REPLI-g than GenomePlex, T > C (7 vs. 1; p = 0.0340), G > T (7 vs. 1; p = 0.0340), G > A (118 vs. 28; p < 0.0001), C > T (76 vs. 15; p < 0.0001), and C > A (12 vs. 3; p = 0.0027; Fig. 5a). Then, we repeated the same analysis including only the germline plus somatic variations. In that case, only the substitution G > A occurred more frequent in REPLI-g than in GenomePlex (21 vs. 4; p < 0.0001, Fig. 5b). However, when we performed the analysis only in false-positive variants created by WGA, we observed that two substitutions were exclusively induced by REPLI-g, G > A (75 vs. 0; P < 0.0001), and C > A (9 vs. 0; p = 0.0027; Fig. 5c). A third substitution also showed significantly higher numbers in REPLI-g C > T (51 vs. 1; p < 0.0001). G > A and C > T base substitutions, also known as C:G > T:A, have been described to account for an important fraction of all the substitution detected in DNA from FFPE tissue specimens^33–36^. Those nucleotide substitutions are considered false positive variants, caused by the cytosine deamination as a consequence of either excessive fixation with formalin^36^ or prolonged incubations of DNA at high temperature^35^. The C:G > T:A base substitutions explained 50.6% of all the altered variants in GenomePlex and 73.1% in REPLI-g. This shows an increased deficiency of REPLI-g in amplifying deaminated cytosines. That REPLI-g’s weakness was even more pronounced in creating false positive variants, since 91.3% of the 138 false positive created by REPLI-g were C:G > T:A substitutions, whereas GenomPlex only created 1 false positive (C > T; Fig. 5c).

## Discussion

Bias is unavoidable when whole genome DNA is amplified^9, 11, 17, 18^ and like most other molecular technologies, every known amplification method has its own drawbacks. For example, DOP-PCR has high error rates, whereas an isothermal or MDA method has the issue of low uniformity^10^. Hybrid methods using both isothermal and PCR amplification seem to perform better in terms of error rates and uniformity^10^. Two hybrid methods (SurePlex and MALAC) showed better results for CNV-seq, a method to detect copy number variation using NGS, than the MDA-based REPLI-g method. Although reports have shown successful results for embryo copy number analysis by array CGH using MDA^17, 37, 38^. However, no single method performs best and satisfies all criteria tested^11^. Moreover, few studies have tested different WGA methods using FFPE cancer specimens and targeted NGS^21^. Most of these used the Illumina sequencers, which are dominant for the NGS market. Yet, the Ion Torrent PGM is still one of the major NGS platforms especially for the small, targeted panels that are increasingly popular and common for genetic analyses in many CLIA laboratories^25, 27–29^. It is, therefore, important to test major WGA methods in cancer FFPE specimens using the Ion Torrent PGM sequencer and targeted NGS panel, as we have done here.

Our study compared the bias introduced by the two most commonly used WGA methods, the MDA-based REPLI-g kit and the hybrid PCR-based GenomePlex kit, with respect to: gene copy number, variation of the VAF values, and the creation of false positive variants during WGA reactions. Our results indicate that non-amplified FFPE showed reliable results for both copy number and variation detection, compared to paired DNA from frozen tissues. FFPE specimens amplified using GenomePlex showed significantly higher copy number bias than non-amplified FFPE samples, but significantly lower copy number and VAF bias than the REPLI-g kit. The MDA-based REPLI-g kit showed very high variations for both copy number and VAF, which suggests that this method should be tested thoroughly and optimized when applied to cancer FFPE specimens using the Ion Torrent PGM and targeted NGS panel.

First, we wanted to exclude any effect caused by FFPE processing, since this is most commonly used in hospitals for various histopathologic diagnosis and other molecular tests. However, formalin affects DNA and RNA integrity and causes mutations in DNA^33, 34^. Thus, we tested whether FFPE samples showed more copy number variations than original frozen samples, and the finding was very minimal differences. Variant allele frequency analysis showed virtually perfect correlations (R^2^ > 0.96) between FFPE and frozen samples for both normal and tumor samples. Thus, we ruled out a major effect caused by FFPE processing for evaluating WGA bias for NGS analyses and results.

Next, we compared results of the two WGA methods, finding a big difference in copy number variation. Bias in the copy number analyses from GenomePlex kit was vastly increased compared to FFPE samples, but was considerably lower than the bias introduced by REPLI-g kit, suggesting that the MDA-based REPLI-g kit cannot be used for copy number analyses in cancer FFPE specimens using targeted NGS without further special optimization.

In the VAF analyses using identified mutations or polymorphisms in non-WGA FFPE tissues, we analyzed the differences in number of altered variants. The pattern was similar. Both kits altered more germline and somatic variants than the non-amplified (non-WGA) FFPE samples. In addition, REPLI-g showed twice as much biased germline and somatic variants than GenomePlex and created in average of 28 false positive variants (created during the WGA), while GenomePlex created 0.2 false positive variants. REPLI-g always had a higher mean VAF bias than GenomePlex. The increment of ΔVAFs in GenomePlex-amplified FFPE DNAs also showed slightly higher but comparable ΔVAFs with non-amplified FFPE DNAs. The mean ΔVAFs from REPLI-g amplified FFPE DNAs was up two times higher ΔVAFs than GenomePlex amplified DNAs.

The use of non-WGA FFPE samples from different tumor types, along with technical replicates of WGA products of the same samples and different library preparation kits, has been a fruitful approach to identify the bias introduced by two more commonly used WGA kits, both at GCN and VAF level. The bias can affect gene dosage, variants call, and its frequencies. Also, it has lead us to speculate that cytosine deamination, caused by excessive formalin fixation^36^, could be the mechanism by which REPLI-g creates 91.3% of the 138 C > T and G > A false positive base substitutions, while GenomePlex just created 1 single false positive. The same mechanism seems to be responsible for the shift of VAF in 50–73% of the germline and/or somatic variants detected WGA products, compared to non-WGA FFPE or frozen DNAs. Although, it was initially difficult to understand how the deaminated cytosines can pose such a challenge to WGA kits and not bias the VAF in non-WGA FFPE DNAs, our findings have been consistent across all the replicates and different samples analyzed, and further studies are warranted.

However, three experimental pieces are missing. First, test of both WGA kits after treatment of FFPE-derived DNA with Uracil-DNA glycosylase, which should revert most of the deaminated cytosines, in theory should help in reducing significantly the C:G > T:A base substitutions^34^. Second, validation of the data with orthogonal methods. Most of the research and clinical laboratories use Sanger sequencing to validate NGS variants. Nonetheless, the National Human Genome Research Institute, recently published a study where 19 out of 5,800 NGS detected variants, were not successfully confirmed by one round of Sanger sequencing^39^. An NGS cross-platform validation would provide a more reliable validation, especially for low frequency variants. Furthermore, it will allow to differentiate platform-specific artifact variants from those strictly created by WGA, with a significant economic cost increase. Third, the use of samples with known genotypes, will also increase the capacity to identify WGA-induced artifacts and platform specific variants. But more importantly, it will be a very useful tool for intra-laboratory validation of any WGA-based NGS assays.

Although the design of our study was intended to minimize the effect of intratumoral heterogeneity in the results, we observed a higher number of biased parameters in the tumor tissues analyzed compare to those obtained in normal tissues. Thus, we cannot rule out that different proportions of tumor cell sub-clones caused the higher rate and range of discrepant variants observed in tumor tissues. Therefore, we think that it is safer to give more weight to the results obtained in normal tissues; this is the strength of our study, which tested both normal and tumor samples using the same testing criteria.

In conclusion, our comparison of two major WGA methods on frozen and FFPE cancer samples suggests that both the MDA-based REPLI-g kit and the hybrid PCR-based GenomePlex kit should undergo significant testing and be optimized for application to cancer FFPE specimens using the Ion Torrent PGM and targeted NGS panel.

## Methods

### Patient Samples and Tissue Specimen Process

Two pairs of normal and tumor tissues from two lung and one esophageal cancer patients were analyzed in the current study. The specimens with informed consents were obtained from participants in the protocol (#11–06107) approved by the Committee for Human Research at the University of California, San Francisco. All experiments were carried out in accordance with the approved guidelines. Tumor and adjacent matched normal lung tissue specimens were collected during surgical procedures and immediately preserved in liquid nitrogen.

H&E stained slides from representative tissue sections were reviewed by a pathologist to ensure the tumor content. Each tissue specimen was cut in half. One half was preserved in liquid nitrogen and the other half was fixed in 10% neutral buffered formalin for 24–36 hours. The fixation was stopped by dehydrating the specimens in ethanol solutions of increased concentration 30%, 50%, 70%, 80%, 95%, and 100% two washes of one hour, each, followed by three washes of Xylene and liquid paraffin, one hour each. After FFPE tissue embedding, a new representative tissue section was stained by H&E and reviewed again by the pathologist to ensure the tissue content.

### DNA Extraction

DNA from frozen tissues was isolated by using DNeasy Blood & Tissue Kit (Qiagen, Valencia, CA) following the manufacturer’s instructions. Briefly, up to 25 mg of tissue was disrupted by using Tissue lyser LT for 5 minutes at 50 Hz. The tissues were completely digested with ATL buffer in presence of Proteinase K at 56 °C for at least one hour. The DNA was purified by using Qiamp MinElute Columns, and eluted in 200 µL of AE buffer.

To extract DNA from FFPE specimens, two to five FFPE sections or a minimum of 10 mm^2^ of FFPE tissue area, containing a minimum of 30% of tumor cells, were deparaffinized in xylene for 10 minutes at room temperature under a dedicated hood. The slides were air dried for 15 minutes. The FFPE tissues were digested in 250 µL of lysis buffer (10 mM Tris HCl pH 8.0; 0.1 mM EDTA; 2% SDS), for 16 hours at 60 °C. Then 10 µL of 20 mg/mL Proteinase K (Qiagen) were added and tissues were incubated for additional 15 minutes at 60 °C. The DNA was extracted with 350 µL of Phenol:Chloroform:Isoamyl alcohol 25:24:1 saturated with 10 mM Tris, pH 8.0, 1 mM EDTA (Sigma Aldrich). The aqueous phase, containing the DNA, was precipitated by centrifugation at room temperature for 15 min. at 12,600 × g, with 2.5 volumes of 100% ethanol in the presence of 0.3 M of NaAc. The DNA pellet was washed with 70% ethanol, air dried and resuspended in 50 µL of molecular biology grade 10 mM Tris HCl pH 8.0; 0.1 mM EDTA solution. The DNA concentration and purity was assessed spectrophotometrically (Nanodrop 8000; Thermo Fisher Scientific). A second DNA concentration quantitation was performed fluorometrically.

### DNA Quantitation

Both, extracted DNA or WGA products were quantified by fluorometry by using Quant-iT™ PicoGreen dsDNA Assay Kit (Thermo Fisher Scientific) as follows: 2 µL of stock DNA were diluted 1:100 v/v in 1 × TE, containing 1:100 diluted Picogreen reagent in a final reaction volume of 40 µL per reaction. Human genomic DNA (Bioline, Taunton, MA), was serially diluted to construct a standard curve ranging from 3 ng/µL to 0.09 ng/µL of DNA. Each experimental sample or dilution point of the standard curve was assayed in triplicate. The fluorometric quantitation was performed in the Synergy HTX platform (Biotek Instruments, Winooski, VT). The DNA was diluted, to a final concentration of 2 ng/µL.

### Experimental Design and WGA Methods

The current study is aimed to determine the impact of two WGA methods in biasing GCN and VAF in NGS data from FFPE cancer specimens. For this purpose, we analyzed three patients sample set. Each sample set is composed by one pair of normal and tumor DNA derived from frozen tissues (experimental condition #1), and its FFPE tissue counterparts (non-WGA FFPE samples; experimental condition #2). DNA from Non-WGA FFPE normal and tumor tissues was used to prepare WGA products using two different kits: first, the GenomePlex® Complete Whole Genome Amplification Kit (experimental condition #3; Sigma-Aldrich, St. Louis, MO, USA; Cat. No. WGA2–50RXN;) and second, REPLI-g FFPE kit (experimental condition #4; Qiagen, Valencia, CA, USA; Cat. No. 150243;) following manufacturer’s instructions. Briefly, for the GenomePlex WGA method, 100 ng of non-WGA FFPE DNA was fragmented by incubating at 95 °C for 4 minutes in 1 × fragmentation buffer followed by ligating universal PCR adaptors. The products were amplified by PCR in a reaction, with an initial DNA denaturation of 3 minutes at 95 °C, 14 PCR cycles with a thermal profile of 95 °C for 15 seconds, and a combined annealing and elongation step of 5 minutes at 65 °C per cycle. In the REPLI-g WGA method, 100 ng of non-WGA FFPE DNA specimens were ligated to produce high molecular weight products, followed by isothermal DNA amplification for two hours. The WGA products from both methods were stored at −20 °C.

To get a better sense on how the inter-experimental variation during the NGS library preparation process affected the bias introduced by WGA, we prepared NGS libraries from one full set of lung cancer sample #1 using the AmpliSeq custom panel in three independent experiments in three consecutive days.

### Setup of VAF Variability Threshold in NGS

To perform an accurate analysis of the VAF bias caused by WGA in DNA derived from FFPE, we first needed to understand the variability introduced in the VAF by the NGS library preparation protocol. Ideally this would be done using DNA extracted from frozen tissues. However, because the REPLI-g kit used in the current study, it is explicitly optimized to amplify FFPE DNA, and it showed an insufficient performance with DNA extracted from frozen tissue (see subheading “*WGA variability in Frozen Tissue*” under *results* section). We studied the technical variability introduced during the NGS library preparation in the independent triplicated NGS libraries from non-WGA FFPE samples (normal and tumor) in sample LC#1. After completing the variant calling (as described below), we calculated the VAF average and standard deviation (SD) for each germline and somatic variant found in each of the replicates from non-WGA FFPE normal and tumor samples. Next, the average of all the SD from VAF was calculated. Among the 14 germline and 11 somatic variants consistently found in all three replicates, we found an average of standard deviation of 0.0316 and 0.0219 in normal and tumor sample, respectively. Thus, we concluded that using a VAF threshold of 0.05 to measure the variability would be a safe and logic value.

### AmpliSeq-DNA NGS Library Preparation

A custom, single pool, multiplexed, PCR-based, NGS library panel was designed using Ion AmpliSeq designer software (Thermo Fisher Scientific, Waltham, MA, USA) to target 97 specific regions from 94 genes for a total panel size of 15.58 Kb per library^34^. The information on the genomic coordinates of the amplicons included in the panel can be found in Supplementary Table 1. The Libraries were constructed using Ion AmpliSeq^TM^ Library Kit v2.0 (Life Technologies), according to the manufacturer’s instructions^34^. To simply explain, 10 ng of DNA from either frozen, non-WGA FFPE or FFPE WGA products were amplified for 21 cycles, followed by FUPA treatment and ligation of Ion Xpress plus Universal adaptors. The libraries were purified with Agentcourt Ampure XP beads (Beckman Coulter, Fullerton, CA) and re-suspended in 1× low TE buffer. The libraries were quantified by using the Ion Library TaqMan™ Quantitation Kit (Thermo Fisher Scientific; Cat. No. 4468802).

### NextDay Seq DNA NGS Libraries Preparation

To study if the bias induced by WGA in NGS, was panel specific, we prepared NGS libraries using a commercially available library preparation kit, which uses a different chemistry than AmpliSeq library preparation kits (NextDay Seq-Pan Cancer HotSpot Panel kit; CureSeq, Inc.). As template, DNA from tumor and normal tissues from both frozen and FFPE specimens (from the esophageal cancer patient) was used as described elsewhere^25^. Briefly, 10 ng of DNA or WGA product were amplified by PCR for 22 cycles, targeting 74 amplicons from 25 genes (*ABL1, AKT1, ALK, BRAF, CTNNB1, DDR2, DNMT3A, EGFR, ERBB2, ESR1, FLT3, GNA11, GNAQ, HRAS, IDH1, IDH2, KRAS, MAP2K1, NRAS, PIK3CA, PTEN, RET, SMAD4, SMO and TSC1)*. Next, universal adaptors and barcodes were ligated to PCR products. Then, the purified libraries (by using a beads-based approach) were eluted in 30 µL. The quality and quantity of the libraries was evaluated by high resolution electrophoresis using the High Sensitivity DNA kit (Agilent Technologies, Santa Clara, CA, USA; cat. no. 5067–4626) and the Bioanalyzer 2100 platform (Agilent Technologies) software.

### Emulsion PCR and Ion Torrent Sequencing

Based on the qPCR quantification of libraries, equimolar amounts of each library were pulled for a final combined molarity of 400 pM. The emPCR was run by using the Ion PGM Template OT2 200 Kit (Thermo Fisher Scientific) and loaded in the Ion OneTouch™ 2 instrument. Next, non-templated Ion Sphere Particles (ISP) beads were eliminated by magnetic bead purification. The mixture of barcoded libraries was sequenced with the Ion PGM Sequencing 200 Kit V2 (Thermo Fisher Scientific). The sequencing beads were loaded in Ion 318 v2 or 316 v2 Chips (Thermo Fisher Scientific), and sequenced on the Ion Torrent™ PGM sequencer (Thermo Fisher Scientific).

### NGS Data Analysis

The data from all the samples were analyzed by using sorted and aligned bam files generated by the Ion Torrent Suite pipeline and the hg19 version of the Human genome as reference. Two different variant callers were used: the plugin included in the Ion Torrent Suite v5.02 (using default parameters) and Samtools mpileup v1.31. The vcf files obtained with Samtools were intersected against the bed file containing the genomic coordinates of the targeted regions of the AmpliSeq custome panel. Only variants that contained DP4 values^40^, a sequencing depth of at least 50× (including both reference and alternative alleles) and a VAF of > 0.04, were used for analysis. Each variant from either pipeline was manually reviewed on Integrated Genomics Viewer (IGV v2.3.85) to: first, filter out false positives (i.e. homopolymer regions and misaligned sequences), and second to check the base and mapping quality of the variants. The sequencing depth and uniformity was analyzed using the Ion Reporter plugin.

### Analysis of GCN

To study the bias introduced by WGA kits at GCN level, an accurate normalization of the PCR-based NGS sequencing data is needed. The normalization of the GCN data in the current study, we used a similar method used in the MLPA technique^41^, but using sequencing depth as value of analysis. The normalization use comprised two steps: an intra-sample, followed by an inter-sample normalization. For the intra-sample normalization, we prepared a spreadsheet with three tabs: one for the reference sample (either non-WGA FFPE normal or tumor data, depending on the tissue type analyzed), and two additional tabs for its paired GenomePlex and REPLI-g samples. Every tab contains a table with *n* + *1* columns and *n* rows, where *n* is the number of amplicons targeted by the sequencing panel. In the first column of each tab, it introduces the sequencing depth of each amplicon (sorted by descendent chromosome number and position). Each of the following columns contains the ratios of one amplicon’s sequencing depth against each of the other 96 amplicons in the case of the AmpliSeq customized panel, or 73 amplicons in the case of the NextDay Seq PAN cancer panel. Next, the average of the ratios (AVRT), from each column is calculated (excluding the ratio of the amplicon against itself). The final GCN value is obtained by dividing the AVRT from one column of the test sample between the AVRT value of the same column from the reference sample. A GCN value of 1 means that the targeted amplicon in the test sample is diploid (if the reference sample is verified to be a diploid sample). If the GCN value is 0.5, the test sample has lost one of the alleles, while if the GCN value is 2 it means that the gene dosage of the targeted amplicon in the tested sample has twice the ploidy of the reference sample.

### Analysis of Variant Allele Frequency (VAF)

The correlation between VAFs and WGA-tumor vs WGA-normal, non-WGA FFPE tumor vs WGA-tumor, and non-WGA FFPE normal vs WGA-normal tissue DNA. 3). For each variant called, we calculated the difference between the VAF found in the non-WGA FFPE reference DNA sample (tumor or normal) and the VAF of the same variant found in the matched DNAs from each of the two experimental conditions (GenomePlex or REPLI-g). Those variants with a difference in VAF (ΔVAF) higher or lower than ± 0.10 were considered to be the variants with “biased” VAFs. The extent of the bias was evaluated by plotting the range of ΔVAF of biased variants for each experimental condition.

### Statistical Analysis

VAF was analyzed as a continuous variable. Each variant was tested for Gaussian distribution using three different normality tests: D’Agostino & Pearson, Shapiro-Wilk, and KS normality tests (GraphPad Prism 6.0 software). The difference in number biased variants, between means of ΔVAF ranges, and between different experimental conditions was assessed by using the two-tailed Mann-Whitney test (comparing ranks). To assess significance in the difference of GCN values range, the two-tailed, Wilcoxon matched-pairs signed rank test was used. The test described elsewhere^42^, was used to study the statistical difference in the number of different base substitutions between GenomePlex and REPLI-g. The *P* values associated with z values were obtained from http://graphpad.com/quickcalcs/PValue1.cfm. A two-tailed, paired t test was used to assess the statistical difference in coverage uniformity. A Wilcoxon matched-pairs signed rank test was used to analyze the mean sequencing depth between different experimental conditions. All the statistical tests were considered significant when α < 0.05.

## Electronic supplementary material

Supplementary Information
