# Supplementary material for: Systematic comparison of two whole-genome amplification methods for targeted next-generation sequencing using frozen and FFPE normal and cancer tissues
Source: Sci Rep. 2017 Jun 22;7:4055. doi: 10.1038/s41598-017-04419-9 (PMC5481435; doi:10.1038/s41598-017-04419-9)
Supplement: Supplementary file 1 — Supplementary Information [file 41598_2017_4419_MOESM1_ESM.docx]

**Supplementary Information**

**Systematic comparison of two whole-genome amplification methods for targeted next-generation sequencing using frozen and FFPE normal and cancer tissues**

Pedro Mendez^1^, Li Tai Fang^1^, David M. Jablons^1,2,*^, and Il-Jin Kim^1,2,*^

^1^Thoracic Oncology Laboratory, Department of Surgery, ^2^Comprehensive Cancer Center, University of California San Francisco, San Francisco, CA, USA,

Correspondence and requests for materials should be addressed to I.J.K (email: [Il-Jin.Kim@ucsf.edu](mailto:Il-Jin.Kim@ucsf.edu)) or D.M.J (email: [David.Jablons@ucsf.edu](mailto:David.Jablons@ucsf.edu))

Key words: Next-generation sequencing (NGS), Whole Genome Amplification (WGA), cancer

**Supplementary Figures**

**Figure S1. Effect of Whole Genome Amplification (WGA) kits in Sequencing coverage uniformity and depth.** Detailed analysis of the (a) Sequencing coverage uniformity and (b) depth values by sample type and WGA kit. The data includes the triplicates from lung cancer #1, the one replicate from the lung cancer #2 and esophageal cancer #1 prepared with the AmpliSeq custom library preparation kit, as well as the esophageal cancer #1 prepared with the NextDay Seq libraby preparation kit. The statistical significance of the coverage uniformity was assessed by a two-tailed, paired *t* test, while the sequencing depth was analyzed by a two-tailed, Wilcoxon matched-pairs signed rank test. * indicates p<0.05, ** p<0.01, *** p<0.001 and **** p<0.0001. FFPE: formalin-fixed and paraffin embedded tissue, WGA: Whole genome amplification.

**Figure S2. NGS libraries yield analyzed by library preparation kit, tissue type and WGA kit.** DNA extracted from FFPE or frozen tissues were amplified with both WGA kits, followed by preparation of the NGS libraries with either (a) AmpliSeq custom or (b) NextDay Seq library preparation kit. The histograms show the value of yield of NGS libraries (pmol/L), of the targeted fragments, calculated from high-resolution electropherograms. FFPE: formalin-fixed and paraffin embedded tissue, WGA: Whole genome amplification.

**Figure S3. Gene Copy Number (GCN) data from AmpliSeq custom library preparation panel in lung cancer #2 from both WGA-amplified FFPE DNAs.**

(**a**) Histogram of GCN values for all 97 amplicons of the AmpliSeq custom library preparation panel in the GenomePlex FFPE-N, (**b**) GenomePlex FFPE-T, (**c**) REPLI-g FFPE-N, (**d**) REPLI-g FFPE-T samples. Each bar represents the GCN value. The range of normal gene dosage is 1 ± 0.25. The normalization of GCN data is described in the Methods section. GCN: normalized gene copy number; FFPE: formalin-fixed and paraffin embedded tissue.

**Figure S4. GCN data from AmpliSeq custom library preparation panel in esophageal cancer #1 from both WGA-amplified FFPE DNAs.**

(**a**) Histogram of GCN values for all 97 amplicons of the AmpliSeq custom library preparation panel in the GenomePlex FFPE-N, (**b**) GenomePlex FFPE-T, (**c**) REPLI-g FFPE-N, (**d**) REPLI-g FFPE-T samples. Each bar represents the GCN value. The range of normal gene dosage is 1 ± 0.25. The normalization of GCN data is described in the Methods section. GCN: normalized gene copy number; FFPE: formalin-fixed and paraffin embedded tissue.

**Figure S5. Range of GCN values across all the experimental conditions from different samples and library preparation panels**. Boxplot (min. to max. and median values) of the range of GCN values of the amplicons included in the library reparation of the AmpliSeq custom panel for the (a) lung cancer case #2, (b) esophageal cancer and (c) esophageal cancer sample prepared with the NextDay Seq panel. A two-tailed, Wilcoxon matched-pairs signed rank test was used to assess the statistical significance of the difference between different experimental conditions. The range of normal gene dosage is 1 ± 0.25. * indicates p<0.05, ** p<0.01, *** p<0.001 and **** p<0.0001. FFPE: formalin-fixed and paraffin embedded tissue, WGA: Whole genome amplification, GCN: Gene copy number.

**Figure S6. GCN data from NextDay Seq library preparation panel in esophageal cancer #1 from both WGA-amplified FFPE DNAs.**

(**a**) Histogram of GCN values for all 74 amplicons of the NextDay Seq library preparation panel (CureSeq Inc.) in the GenomePlex FFPE-N, (**b**) GenomePlex FFPE-T, (**c**) REPLI-g FFPE-N, (**d**) REPLI-g FFPE-T samples. Each bar represents the GCN value. The range of normal gene dosage is 1 ± 0.25. The normalization of GCN data is described in the Methods section. GCN: normalized gene copy number; FFPE: formalin-fixed and paraffin embedded tissue.

**Supplementary Table 1.** Targeted NGS panel amplicons of the AmpliSeq custom panel

| Amplicon No. | Chr. # | Start | End | amplicon length (bp) | %GC content |
| --- | --- | --- | --- | --- | --- |
| 1 | chr1 | 21268382 | 21268559 | 177 | 36.0 |
| 2 | chr1 | 62728747 | 62728922 | 175 | 59.1 |
| 3 | chr1 | 75055326 | 75055507 | 181 | 44.5 |
| 4 | chr1 | 89652014 | 89652177 | 163 | 47.0 |
| 5 | chr1 | 110593506 | 110593655 | 149 | 48.7 |
| 6 | chr1 | 110883597 | 110883723 | 126 | 55.1 |
| 7 | chr1 | 150915295 | 150915467 | 172 | 44.5 |
| 8 | chr1 | 162741780 | 162741957 | 177 | 60.1 |
| 9 | chr1 | 177915529 | 177915708 | 179 | 57.8 |
| 10 | chr1 | 182554624 | 182554803 | 179 | 53.9 |
| 11 | chr1 | 246810528 | 246810671 | 143 | 59.7 |
| 12 | chr1 | 247150500 | 247150667 | 167 | 38.7 |
| 13 | chr2 | 29519728 | 29519853 | 125 | 57.9 |
| 14 | chr2 | 37450475 | 37450648 | 173 | 36.8 |
| 15 | chr2 | 37455299 | 37455474 | 175 | 42.0 |
| 16 | chr2 | 54884932 | 54885087 | 155 | 59.0 |
| 17 | chr2 | 61712876 | 61713050 | 174 | 35.4 |
| 18 | chr2 | 70528003 | 70528177 | 174 | 60.0 |
| 19 | chr2 | 223806134 | 223806307 | 173 | 37.9 |
| 20 | chr2 | 225266137 | 225266310 | 173 | 58.0 |
| 21 | chr3 | 123444809 | 123444934 | 125 | 63.5 |
| 22 | chr3 | 134911515 | 134911700 | 185 | 52.7 |
| 23 | chr4 | 38828739 | 38828877 | 138 | 46.0 |
| 24 | chr4 | 88767480 | 88767657 | 177 | 53.4 |
| 25 | chr4 | 110459624 | 110459791 | 167 | 35.7 |
| 26 | chr4 | 128807554 | 128807725 | 171 | 40.1 |
| 27 | chr4 | 190878531 | 190878677 | 146 | 35.4 |
| 28 | chr5 | 66416851 | 66417014 | 163 | 34.8 |
| 29 | chr5 | 109125133 | 109125281 | 148 | 28.2 |
| 30 | chr5 | 112173515 | 112173686 | 171 | 39.5 |
| 31 | chr5 | 130791566 | 130791746 | 180 | 36.5 |
| 32 | chr5 | 130841175 | 130841306 | 131 | 26.5 |
| 33 | chr5 | 154304041 | 154304188 | 147 | 40.5 |
| 34 | chr6 | 30918071 | 30918173 | 102 | 52.4 |
| 35 | chr6 | 31379682 | 31379841 | 159 | 60.0 |
| 36 | chr6 | 32629049 | 32629210 | 161 | 56.8 |
| 37 | chr6 | 46676972 | 46677104 | 132 | 27.1 |
| 38 | chr6 | 117631280 | 117631436 | 156 | 45.2 |
| 39 | chr6 | 129634030 | 129634199 | 169 | 47.1 |
| 40 | chr6 | 149983093 | 149983269 | 176 | 37.3 |
| 41 | chr7 | 5567857 | 5568033 | 176 | 63.3 |
| 42 | chr7 | 27934948 | 27935096 | 148 | 50.3 |
| 43 | chr7 | 36660298 | 36660474 | 176 | 40.7 |
| 44 | chr7 | 57529027 | 57529199 | 172 | 40.5 |
| 45 | chr7 | 75211330 | 75211499 | 169 | 57.1 |
| 46 | chr7 | 105278789 | 105278872 | 83 | 52.4 |
| 47 | chr7 | 120385758 | 120385917 | 159 | 35.6 |
| 48 | chr8 | 38147990 | 38148168 | 178 | 46.4 |
| 49 | chr8 | 38172888 | 38173052 | 164 | 43.6 |
| 50 | chr9 | 19786174 | 19786342 | 168 | 46.7 |
| 51 | chr9 | 21206951 | 21207129 | 178 | 49.7 |
| 52 | chr9 | 33385659 | 33385820 | 161 | 59.9 |
| 53 | chr9 | 107367445 | 107367617 | 172 | 53.2 |
| 54 | chr9 | 131387359 | 131387501 | 142 | 48.3 |
| 55 | chr9 | 135800877 | 135801052 | 175 | 34.7 |
| 56 | chr10 | 20534359 | 20534515 | 156 | 41.4 |
| 57 | chr10 | 24874511 | 24874689 | 178 | 53.1 |
| 58 | chr10 | 101162381 | 101162552 | 171 | 48.3 |
| 59 | chr11 | 4471230 | 4471402 | 172 | 51.4 |
| 60 | chr12 | 25380179 | 25380336 | 157 | 41.1 |
| 61 | chr12 | 25398241 | 25398365 | 124 | 36.8 |
| 62 | chr12 | 30862937 | 30863111 | 174 | 42.9 |
| 63 | chr12 | 118671360 | 118671533 | 173 | 27.6 |
| 64 | chr12 | 120139603 | 120139772 | 169 | 51.8 |
| 65 | chr12 | 121431382 | 121431564 | 182 | 60.1 |
| 66 | chr13 | 21557575 | 21557745 | 170 | 53.8 |
| 67 | chr13 | 25670914 | 25671090 | 176 | 41.2 |
| 68 | chr13 | 111857551 | 111857730 | 179 | 38.9 |
| 69 | chr14 | 25044390 | 25044560 | 170 | 56.1 |
| 70 | chr15 | 40309302 | 40309429 | 127 | 46.1 |
| 71 | chr16 | 50744445 | 50744629 | 184 | 56.2 |
| 72 | chr17 | 4645027 | 4645196 | 169 | 61.8 |
| 73 | chr17 | 10434950 | 10435127 | 177 | 42.7 |
| 74 | chr17 | 20768692 | 20768788 | 96 | 33.0 |
| 75 | chr17 | 39155917 | 39156097 | 180 | 63.5 |
| 76 | chr17 | 41886373 | 41886503 | 130 | 32.1 |
| 77 | chr17 | 80615672 | 80615853 | 181 | 58.8 |
| 78 | chr18 | 32917542 | 32917714 | 172 | 41.6 |
| 79 | chr18 | 70417263 | 70417436 | 173 | 46.0 |
| 80 | chr19 | 10076931 | 10077047 | 116 | 57.3 |
| 81 | chr19 | 12881980 | 12882145 | 165 | 64.5 |
| 82 | chr19 | 20229781 | 20229916 | 135 | 39.7 |
| 83 | chr19 | 38610160 | 38610340 | 180 | 57.5 |
| 84 | chr19 | 40520377 | 40520549 | 172 | 38.2 |
| 85 | chr19 | 56274392 | 56274559 | 167 | 43.5 |
| 86 | chr19 | 58199225 | 58199355 | 130 | 44.3 |
| 87 | chr19 | 58452918 | 58453093 | 175 | 42.6 |
| 88 | chr21 | 11049526 | 11049690 | 164 | 36.4 |
| 89 | chr21 | 14982810 | 14982979 | 169 | 58.2 |
| 90 | chr21 | 19653344 | 19653447 | 103 | 33.7 |
| 91 | chr22 | 25019742 | 25019925 | 183 | 63.6 |
| 92 | chrX | 15403074 | 15403253 | 179 | 41.1 |
| 93 | chrX | 21458805 | 21458950 | 145 | 34.9 |
| 94 | chrX | 48672858 | 48673037 | 179 | 60.6 |
| 95 | chrX | 53115027 | 53115214 | 187 | 44.7 |
| 96 | chrX | 83128710 | 83128842 | 132 | 36.1 |
| 97 | chrX | 142718761 | 142718886 | 125 | 42.1 |
